# Do All Components of Psychological Wellbeing Predict Cognitive Function?

**DOI:** 10.1093/geroni/igaf122.050

**Published:** 2025-12-31

**Authors:** Karysa Britton, Gabrielle Pfund, Emily Willroth

**Affiliations:** Washington University in St. Louis, St. Louis, Missouri, United States; Auburn University, Auburn, Alabama, United States; Washington University in St. Louis, St. Louis, Missouri, United States

## Abstract

Psychological wellbeing, specifically sense of purpose, has been consistently associated with cognitive functioning and dementia. Less is known about associations between other components of psychological wellbeing and cognitive functioning, an indicator of dementia. Given the importance of identifying modifiable risk and protective factors for dementia, the current study assessed whether components of psychological wellbeing are cross-sectionally and prospectively associated with cognitive function and cognitive change. A total of 4,026 participants from the Midlife in the United States study were included in analyses (MAge=56.18, SD = 12.38). The sample was representative of both males (45%) and females (55%), individuals primarily identified as White (93%) followed by Black or African American (4%), and about half of the participants reported that they completed an associate’s degree or higher (45%). Components of wellbeing were assessed with the Ryff Psychological Wellbeing Scales (1989) and cognition was assessed with the Brief Test of Adult Cognition. All six components of wellbeing were significantly and positively associated with cognition cross-sectionally and effect sizes ranged from small to medium across components (βs=0.07-0.19) with personal growth having the largest association. Moreover, higher levels of environmental mastery, personal growth, sense of purpose, and self-acceptance significantly predicted increased levels of cognition at a nine-year follow-up when controlling for cognition at the first assessment timepoint (β = 0.03-0.04]). These findings extend past wellbeing research by demonstrating that in addition to sense of purpose, some components of psychological wellbeing but not others are also associated with cognitive outcomes and should be assessed as possible protective factors for ADRD.

